# Supplementary material for: False Negative Mitigation in Group Testing for COVID-19 Screening
Source: Front Med (Lausanne). 2021 May 10;8:661277. doi: 10.3389/fmed.2021.661277 (PMC8170512; doi:10.3389/fmed.2021.661277)
Supplement: Supplementary file 1 [file Data_Sheet_1.PDF]

False Negative Mitigation in Group Testing for COVID-19 Screening

A. R. Alizad-Rahvar*, S. Vafadar†, M. Totonchi††, M. Sadeghi**
*School of Biological Sciences, Institute for Research in Fundamental Sciences (IPM), Tehran, Iran
†Laboratory of Biological Complex Systems and Bioinformatics (CBB), Institute of Biochemistry and Biophysics, University of Tehran, Tehran, Iran
††Department of Genetics, Royan Institute for Reproductive Biomedicine, ACECR, Tehran, Iran
**National Institute for Genetic Engineering and Biotechnology, Tehran, Iran
Corresponding authors' email: *alizad@ipm.ir, **sadeghi@nigeb.ac.ir

I. Analysis

In our analysis, we present S_g, and the average number of tests per sample, i.e., $\overline{T}/N$, for each method. Then, we explain how to obtain n_{opt}. First, we introduce some notations and basic equations to prove our analysis accordingly.

- $\#(x) \triangleq$ the number of the occurrence of event x
- $E[X] \triangleq$ the expected value of a random variable X
- $m \triangleq \#(\text{groups}) = N/n$ (m may not equal an integer, however, we do not round it up in our analysis, so that we can calculate the derivatives and simplify the equations. We will round up the all integer size values at the end of our analysis).
- TP_g: the detected positive group is a true positive
- FP_g: the detected positive group is a false positive
- The probability of having a positive group is

$$P_g \triangleq P(G=1) = 1 - P(G=0) = 1 - (1-p)^n.$$

- Group-level sensitivity (true-positive rate TPR_g):

$$S_g \triangleq P(\hat{G}=1 \mid G=1) = \frac{P(\hat{G}=1, G=1)}{P(G=1)} = \frac{P(\text{TP}_g)}{P_g}.$$

  This equation yields $P(\text{TP}_g) = P_g S_g$.
- Group-level FPR:

$$\text{FPR}_g \triangleq P(\hat{G}=1 \mid G=0) = \frac{P(\hat{G}=1, G=0)}{P(G=0)} = \frac{P(\text{FP}_g)}{1-P_g}.$$

  Hence, $P(\text{FP}_g) = (1-P_g)\ \text{FPR}_g$.
- $P_g^+ \triangleq P(\hat{G}=1) = P(\text{TP}_g) + P(\text{FP}_g) = S_g P_g + (1-P_g)\ \text{FPR}_g$.
- $n_g^+ \triangleq E[\#(\text{groups diagnosed positive})] = m P_g^+$.
- Calculation of $\overline{T}/N$ for 1-stage group-test methods:

$$\begin{aligned} \overline{T} \triangleq E[T] &= \#(\text{group tests}) + E[\#(\text{individual tests})] \\ &= \#(\text{tests on each group}) \times m + E[\#(\text{groups diagnosed positive})] \times n \\ &= rm + n_g^+ n \\ &\Rightarrow \frac{\overline{T}}{N} = \frac{r}{n} + P_g^+, \end{aligned} \tag{1}$$

  where $r = 1$ for the groupMix and the conventional methods, and $r = 2$ for the 2Rgt method.
- $\hat{M}_{i,i+1}$: the test result of the mixed group $M_{i,i+1}$

A. Analysis of the conventional group-test method

1) S_g:

$$\begin{aligned} S_g^{\text{conv}} &= P(\hat{G} = 1 \mid G = 1) \\ &= 1 - P(\hat{G} = 0 \mid G = 1) \\ &= 1 - f_N. \end{aligned}$$

2) $\overline{T}/N$: According to Eq. (1) we have

$$\begin{aligned} \frac{\overline{T}_{\text{conv}}}{N} &= \frac{1}{n} + P_g S_g^{\text{conv}} \\ &= \frac{1}{n} + (1 - f_N) P_g. \end{aligned}$$

B. Analysis of the 2Rgt method

Assume that $\hat{G}^{(1)}$ and $\hat{G}^{(2)}$ are the results of the first and the second test of G. $\hat{G}^{(1)}$ and $\hat{G}^{(2)}$ are dependent because they both depend on whether G is infected or not. However, they are conditionally independent given G; i.e.,

$$P(\hat{G}^{(1)}, \hat{G}^{(2)} \mid G) = P(\hat{G}^{(1)} \mid G) P(\hat{G}^{(2)} \mid G).$$

1) S_g:

$$\begin{aligned} S_g^{\text{2Rgt}} &= P(\hat{G} = 1 \mid G = 1) \\ &= 1 - P(\hat{G} = 0 \mid G = 1) \\ &= 1 - P(\hat{G}^{(1)} = 0, \hat{G}^{(2)} = 0 \mid G = 1) \\ &= 1 - P(\hat{G}^{(1)} = 0 \mid G = 1) P(\hat{G}^{(2)} = 0 \mid G = 1) \\ &= 1 - f_N^2. \end{aligned}$$

2) $\overline{T}/N$: According to Eq. (1) we have

$$\begin{aligned} \frac{\overline{T}_{\text{2Rgt}}}{N} &= \frac{2}{n} + P_g S_g^{\text{2Rgt}} \\ &= \frac{2}{n} + (1 - f_N^2) P_g. \end{aligned}$$

C. Analysis of the 2S-2Rgt method

1) S_g: Assume that $\hat{G}^{(1)}$ and $\hat{G}^{(2)}$ are the test results of G in the first and the second stage of the sequential 2Rgt (2S-2Rgt) method.

$$\begin{aligned} S_g^{\text{2S-2Rgt}} &= P(\hat{G} = 1 \mid G = 1) \\ &= P(\hat{G}^{(1)} = 1 \mid G = 1) + P(\hat{G}^{(1)} = 0, \hat{G}^{(2)} = 1 \mid G = 1) \\ &= P(\hat{G}^{(1)} = 1 \mid G = 1) + P(\hat{G}^{(1)} = 0 \mid G = 1) P(\hat{G}^{(2)} = 1 \mid G = 1) \\ &= (1 - f_N) + f_N(1 - f_N) \\ &= 1 - f_N^2. \end{aligned}$$

S_g of the 2S-2Rgt is the same as that of the 2Rgt method.

2) $\overline{T}/N$:

$$\begin{aligned}\overline{T}_{\text{2S-2Rgt}} &= \#(\text{groups}) + \#(\text{groups diagnosed negative in 1st stage}) + E[\#(\text{individual tests})]\\ &= m + m[1 - P_g(1-f_N)] + (n_g^+ \times n)\\ &\Rightarrow \frac{\overline{T}_{\text{2S-2Rgt}}}{N} = [2 - P_g(1-f_N)]\frac{1}{n} + (1-f_N^2)P_g.\end{aligned}$$

The term $2 - P_g(1-f_N)$ on the right-hand side is less than 2; hence, $\overline{T}/N$ in the 2S-2Rgt method is less than that in the non-adptive 2Rgt method. Indeed, we save on average $NP_g(1-f_N)/n$ tests with the sequential approach.

D. ***Analysis of the C-groupMix method***

In the groupMix method, $\hat{M}_{i-1,i}$ and $\hat{M}_{i,i+1}$ are conditionally independent given G_i, i.e.,

$$P(\hat{M}_{i-1,i}, \hat{M}_{i,i+1} \mid G_i) = P(\hat{M}_{i-1,i} \mid G_i)P(\hat{M}_{i,i+1} \mid G_i).$$

Moreover, we use the following conditional probabilities in the proof of our analyses for the groupMix method.

1) $P(\hat{M}_{i-1,i} = 1 \mid G_i = 1) = 1 - f_N$
2) $P(\hat{M}_{i-1,i} = 0 \mid G_i = 1) = f_N$
3) $P(\hat{M}_{i-1,i} = 1 \mid G_i = 0) = P(G_{i-1} = 1)P(\hat{M}_{i-1,i} = 1 \mid G_{i-1} = 1, G_i = 0) = P_g(1-f_N)$
4) $P(\hat{M}_{i-1,i} = 0 \mid G_i = 0) = 1 - P_g(1-f_N)$

The same equations are valid for $\hat{M}_{i,i+1}$.

1) S_g:

$$\begin{aligned}S_g^{\text{C-groupMix}} &= P(\hat{G}_i = 1 \mid G_i = 1)\\ &= 1 - P(\hat{G}_i = 0 \mid G_i = 1)\\ &= 1 - P(\hat{M}_{i-1,i} = 0, \hat{M}_{i,i+1} = 0 \mid G_i = 1)\\ &= 1 - P(\hat{M}_{i-1,i} = 0 \mid G_i = 1)P(\hat{M}_{i,i+1} = 0 \mid G_i = 1)\\ &= 1 - f_N^2.\end{aligned}$$

2) $\overline{T}/N$: There is an algorithmic false positive in the groupMix method. Hence, we should first obtain the $\text{FPR}_g^{\text{C-groupMix}}$ to calculate P_g^+, and then calculate $\overline{T}/N$.

$$\begin{aligned}\text{FPR}_g^{\text{C-groupMix}} &= P(\hat{G}_i = 1 \mid G_i = 0)\\ &= 1 - P(\hat{G}_i = 0 \mid G_i = 0)\\ &= 1 - P(\hat{M}_{i-1,i} = 0, \hat{M}_{i,i+1} = 0 \mid G_i = 0)\\ &= 1 - P(\hat{M}_{i-1,i} = 0 \mid G_i = 0)P(\hat{M}_{i,i+1} = 0 \mid G_i = 0)\\ &= 1 - [1 - P_g(1-f_N)]^2.\end{aligned}$$

Then, by using Eq. (1) we have

$$\begin{aligned}\frac{\overline{T}_{\text{C-groupMix}}}{N} &= \frac{1}{n} + P_g^+\\ &= \frac{1}{n} + P_g S_g^{\text{C-groupMix}} + (1-P_g)\ \text{FPR}_g^{\text{C-groupMix}}\\ &= \frac{1}{n} + (3 - 2f_N - f_N^2)P_g + (-3 + 4f_N - f_N^2)P_g^2 + (1-f_N)^2P_g^3.\end{aligned}$$

E. ***Analysis of the NC-groupMix method***

1) S_g: In the NC-groupMix method, the primary group G_i is detected positive if the test results of both $M_{i-1,i}$ and $M_{i,i+1}$ are positive, or if we have a single positive mixed group, i.e., either $M_{i-1,i}$ or $M_{i,i+1}$. Therefore, we can calculate S_g as follows:

$$\begin{aligned} S_g^{\text{NC-groupMix}} &= P(\hat{G}_i = 1 \mid G_i = 1) \\ &= P(\hat{M}_{i-1,i} = 1, \hat{M}_{i,i+1} = 1 \mid G_i = 1) \\ &+ P(\hat{M}_{i-2,i-1} = 0, \hat{M}_{i-1,i} = 1, \hat{M}_{i,i+1} = 0 \mid G_i = 1) \\ &+ P(\hat{M}_{i-1,i} = 0, \hat{M}_{i,i+1} = 1, \hat{M}_{i+1,i+2} = 0, \mid G_i = 1). \end{aligned}$$

The second and the third probabilities in the above equation are equal. Hence, we have

$$\begin{aligned} S_g^{\text{NC-groupMix}} &= P(\hat{M}_{i-1,i} = 1 \mid G_i = 1)P(\hat{M}_{i,i+1} = 1 \mid G_i = 1) \\ &+ 2P(G_{i-1} = 0)P(\hat{M}_{i-2,i-1} = 0, \hat{M}_{i-1,i} = 1, \hat{M}_{i,i+1} = 0 \mid G_{i-1} = 0, G_i = 1) \\ &+ 2P(G_{i-1} = 1)P(\hat{M}_{i-2,i-1} = 0, \hat{M}_{i-1,i} = 1, \hat{M}_{i,i+1} = 0 \mid G_{i-1} = 1, G_i = 1). \end{aligned}$$

By applying the multiplying property of the conditional independence and using the equations explained at the beginning of Section I-D, we obtain

$$S_g^{\text{NC-groupMix}} = (1 - f_N^2) + 2(P_g^2 - 2P_g)f_N(1 - f_N)^2.$$

2) $\overline{T}/N$: For calculating $\text{FPR}_g^{\text{NC-groupMix}}$, the equations are like the equations for calculating S_g, except that the condition is $G_i = 0$. Ultimately, we obtain

$$\text{FPR}_g^{\text{NC-groupMix}} = P_g^2(1 - f_N)^2 + 2P_g[1 - P_g(1 - f_N)]f_N(1 - f_N).$$

Therefore, we have

$$\begin{aligned} \frac{\overline{T}_{\text{NC-groupMix}}}{N} &= \frac{1}{n} + P_g S_g^{\text{NC-groupMix}} + (1 - P_g)\ \text{FPR}_g^{\text{NC-groupMix}} \\ &= \frac{1}{n} + (1 + 2f_N - 3f_N^2)P_g + (1 - 10f_N + 15f_N^2 - 6f_N^3)P_g^2 + \\ &\quad (-1 + 6f_N - 9f_N^2 + 4f_N^3)P_g^3. \end{aligned}$$

F. ***Analysis of the 2S-groupMix method***

In the 2S-groupMix method, we first perform the NC-groupMix method, and then, the second stage of the test is done based on the results of the first stage. Therefore, the S_g and $\overline{T}/N$ values of the 2S-groupMix method are calculated by using the sum of those values in the NC-groupMix method with the added values obtained by performing the second stage. In the following analyses, the superscript indexes (1) and (2) denote the first and the second stage of the 2S-groupMix method, respectively.

1) S_g:

$$\begin{aligned} S_g^{\text{2S-groupMix}} &= \frac{P(\text{TP}_g)}{P_g} = \frac{P(\text{TP}_g^{(1)}) + P(\text{TP}_g^{(2)})}{P_g} \\ &= P(\hat{G}^{(1)} = 1 \mid G = 1) + P(\hat{G}^{(2)} = 1 \mid G = 1) \\ &= S_g^{\text{NC-groupMix}} + S_g^{(2)}, \end{aligned}$$

where $\text{TP}_g^{(1)}$ and $\text{TP}_g^{(2)}$ mean the true-positive detection of G that is detected in the first and second stages, respectively.

In the second stage, the following situations yield a true positive detection of G_i ($\hat{M}$ values are the test results of the mixed groups in the first stage):

$$\begin{aligned} S_g^{(2)} &= P(\hat{G}_i^{(2)} = 1 \mid G_i = 1) \\ &= P(\hat{M}_{i-2,i-1} = 1, \hat{M}_{i-1,i} = 1, \hat{M}_{i,i+1} = 0, G_{i-1} = 0 \mid G_i = 1) && \text{(I)} \\ &+ P(\hat{M}_{i-2,i-1} = 1, \hat{M}_{i-1,i} = 1, \hat{M}_{i,i+1} = 0, G_{i-1} = 1, \hat{G}_{i-1} = 0 \mid G_i = 1) && \text{(II)} \\ &+ P(\hat{M}_{i-1,i} = 0, \hat{M}_{i,i+1} = 1, \hat{M}_{i+1,i+2} = 1, G_{i+1} = 0 \mid G_i = 1) && \text{(III)} \\ &+ P(\hat{M}_{i-1,i} = 0, \hat{M}_{i,i+1} = 1, \hat{M}_{i+1,i+2} = 1, G_{i+1} = 1, \hat{G}_{i+1} = 0 \mid G_i = 1). && \text{(IV)} \end{aligned}$$

Because of the symmetry, the probabilities (I) and (III), and (II) and (IV) are equal. Therefore, we calculate only the probabilities (I) and (II) separately and then obtain the value of $S_g^{(2)}$.

$$\begin{aligned} \text{(I)}: \quad & P(\hat{M}_{i-2,i-1} = 1, \hat{M}_{i-1,i} = 1, \hat{M}_{i,i+1} = 0, G_{i-1} = 0 \mid G_i = 1) \\ &= P(G_{i-1} = 0) P(\hat{M}_{i-2,i-1} = 1, \hat{M}_{i-1,i} = 1, \hat{M}_{i,i+1} = 0 \mid G_{i-1} = 0, G_i = 1) \\ &= (1 - P_g) P(\hat{M}_{i-2,i-1} = 1 \mid G_{i-1} = 0) P(\hat{M}_{i-1,i} = 1 \mid G_{i-1} = 0, G_i = 1) P(\hat{M}_{i,i+1} = 0 \mid G_i = 1) \\ &= P_g (1 - P_g) f_N (1 - f_N)^2. \end{aligned}$$

Similarly, we have

$$\begin{aligned} \text{(II)}: \quad & P(\hat{M}_{i-2,i-1} = 1, \hat{M}_{i-1,i} = 1, \hat{M}_{i,i+1} = 0, G_{i-1} = 1, \hat{G}_{i-1} = 0 \mid G_i = 1) \\ &= P(G_{i-1} = 1, \hat{G}_{i-1} = 0) P(\hat{M}_{i-2,i-1} = 1, \hat{M}_{i-1,i} = 1, \hat{M}_{i,i+1} = 0 \mid G_{i-1} = 1, G_i = 1) \\ &= f_N (1 - f_N)^2 \times P(G_{i-1} = 1, \hat{G}_{i-1} = 0). \end{aligned}$$

In the above equation, $G_{i-1} = 1$ and $\hat{G}_{i-1} = 0$ means that the primary group G_{i-1} has at least one infected sample, but the individual test results of the infected samples in the first stage are all false negative. Therefore, $P(G_{i-1} = 1, \hat{G}_{i-1} = 0)$ denoted by $\sum(n, p, f_N)$ is as follows:

$$\sum(n, p, f_N) \triangleq P(G_{i-1} = 1, \hat{G}_{i-1} = 0) = \sum_{k=1}^{n} \binom{n}{k} (p f_N)^k (1 - p)^{n-k},$$

where $p f_N$ is the probability of a sample to be infected and diagnosed negative falsely.

Ultimately, we can obtain S_g of the 2S-groupMix method by using the above results.

$$\begin{aligned} S_g^{\text{2S-groupMix}} &= S_g^{\text{NC-groupMix}} + S_g^{(2)} \\ &= (1 - f_N^2) + 2 \left[(1 - 3P_g + P_g^2) + \sum(n, p, f_N) \right] f_N (1 - f_N)^2. \end{aligned}$$

2) $\overline{T}/N$: Similarly, we have

$$\text{FPR}_g^{\text{2S-groupMix}} = \text{FPR}_g^{\text{NC-groupMix}} + \text{FPR}_g^{(2)}.$$

Equations (I) to (IV) used in calculation of $S_g^{(2)}$ can be used for calculating $\text{FPR}_g^{(2)}$ by replacing the condition by $G_i = 0$. After performing the same calculations, we have

$$\text{FPR}_g^{\text{2S-groupMix}} = P_g^2 (1 - f_N)^2 + 2[1 - P_g(1 - f_N)](1 - f_N) \left[P_g f_N + (1 - f_N) \sum(n, p, f_N) \right].$$

Finally,

$$\frac{\overline{T}_{\text{2S-groupMix}}}{N} = \frac{1}{n} + P_g S_g^{\text{2S-groupMix}} + (1 - P_g)\ \text{FPR}_g^{\text{2S-groupMix}}.$$

G. *Finding the optimum group size*

To find the optimum group size n_{opt}, we should find the minimum value of $\overline{T}$ against n. Hence, we need to find the solution of $d\overline{T}/dn = 0$. This solution is the same as the solution of $d(\overline{T}/N)/dn = 0$.

By using Eq. (1) for 1-stage group-testing methods, we have

$$\begin{aligned} &\frac{d(\overline{T}/N)}{dn} = 0 \\ &\Rightarrow \frac{-r}{n^2} + \frac{dP_g^+}{dP_g} \times \frac{dP_g}{dn} = 0, \end{aligned}$$

where

$$\frac{dP_g}{dn} = \frac{-1}{n}(1 - P_g) \times \mathrm{Ln}\,(1 - P_g)\,.$$

n_{opt} can be founf by solving the above equation.

In the case of the 2S-groupMix method, P_g^+ is not only a function of P_g, and the derivative of $\sum(n, p, f_N)$ does not have a closed-form solution. Therefore, for this method, we find n_{opt} by using numerical computation. For the 2S-2Rgt method, the calculation of $d(\overline{T}/N)/dn$ is straightforward when the above formula for dP_g/dn is used. Ultimately, the value of n_{opt} obtained by solving the equation $d(\overline{T}/N)/dn = 0$ should be rounded to the closest integer number.

II. groupMix Web Interface

In this appendix, we explain different parts of the groupMix web interface. In this web, the 1-stage NC-groupMix method is implemented. The following sections in this web interface can be navigated using the left side of the website.

A. *Create Groups*

In the "Create Groups" section (Fig. II.1), we should set three input values of the number of specimens, prevalence (%), and FNR (%). Then, the optimum size of the primary groups, i.e.,n_{opt}, the number of groups, and the average number of tests is calculated according to the provided input

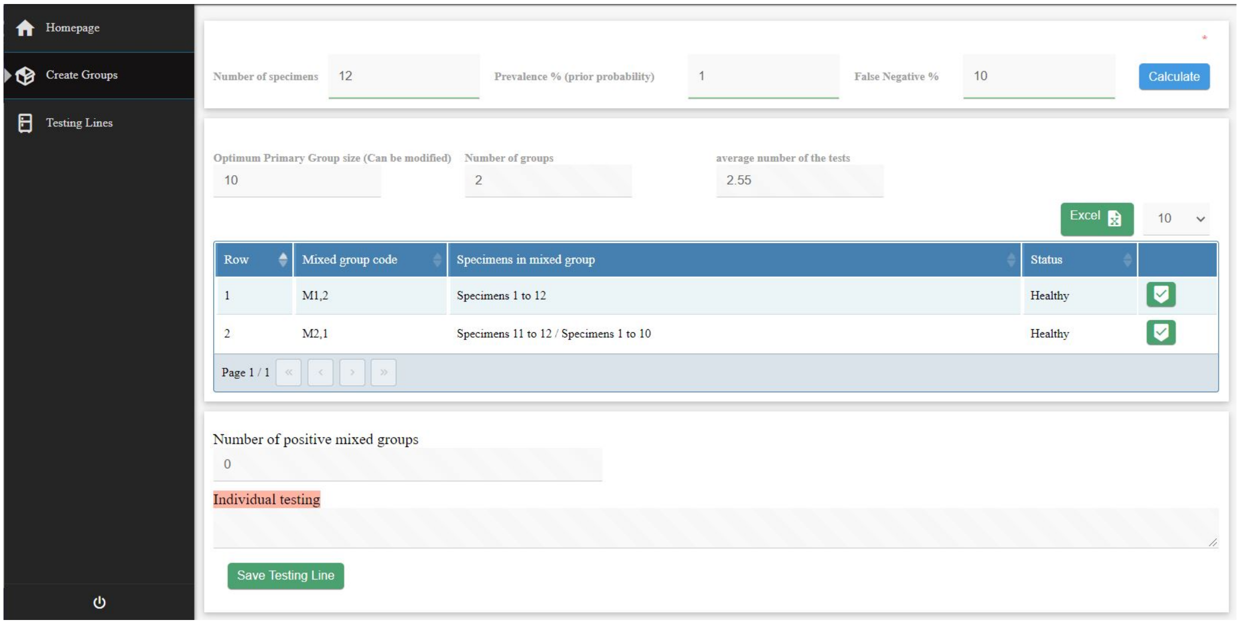

Fig. II.1. "Create Groups" section in the groupMix web interface.

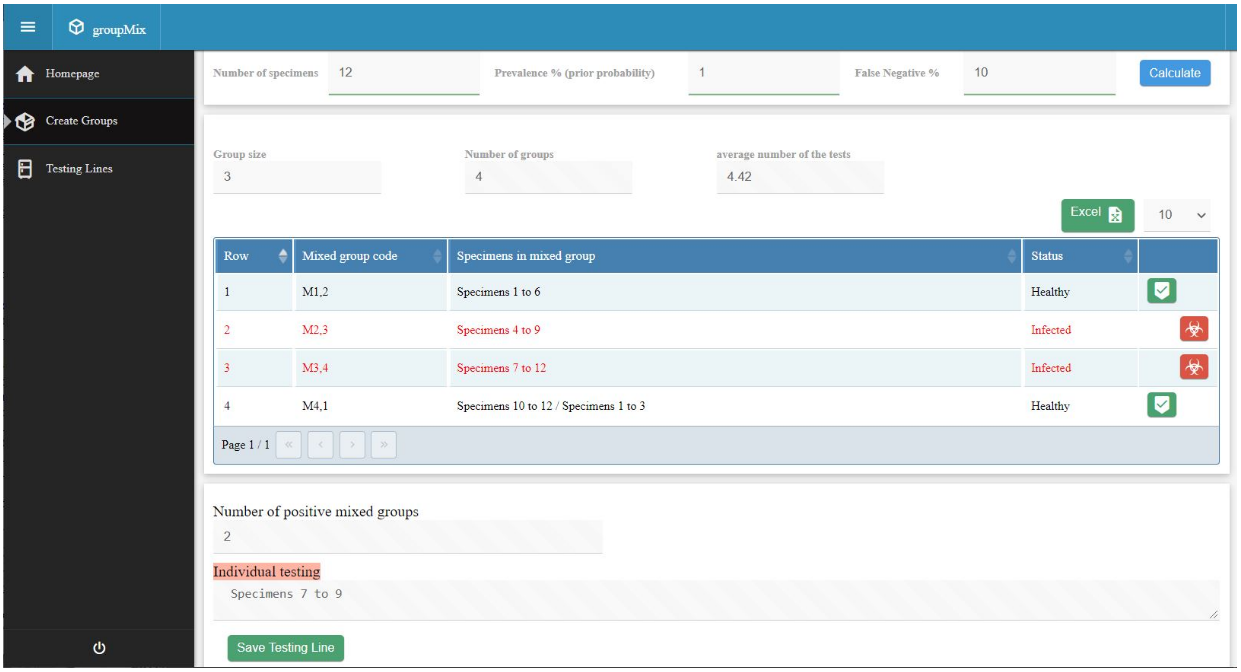

Fig. II.2. "Create Groups" section in the groupMix web interface for $N = 12$, $p = 1\%$, FNR= 10%, and $n = 3$.

values. Under these values, the mixed groups and their corresponding specimen numbers are depicted. The group size can be changed to a different value.

For instance, in Fig. II.2, the input values, and the group size are set according to the case of group testing depicted in Fig. **??**, where we have 12 samples and four mixed groups. After performing the tests on mixed groups, we can set the test results by clicking on the green icons in the "Test Results" column. The green icon indicates that the mixed group is uninfected, and it turns into a red color, indicating infected, by clicking on the green icon. After determining the infected mixed groups, the index of specimens that should be tested by individual testing, i.e., the samples of infected primary groups detected by the non-conservative group detection method, are listed at the bottom of the page. Finally, this test setup can be saved by clicking on the "Save Test Line" button.

B. Test Lines

In this section, we can see the list of saved test lines (Fig. II.3). Moreover, we can edit or delete each test line, and download the list of available test lines as an Excel spreadsheet.

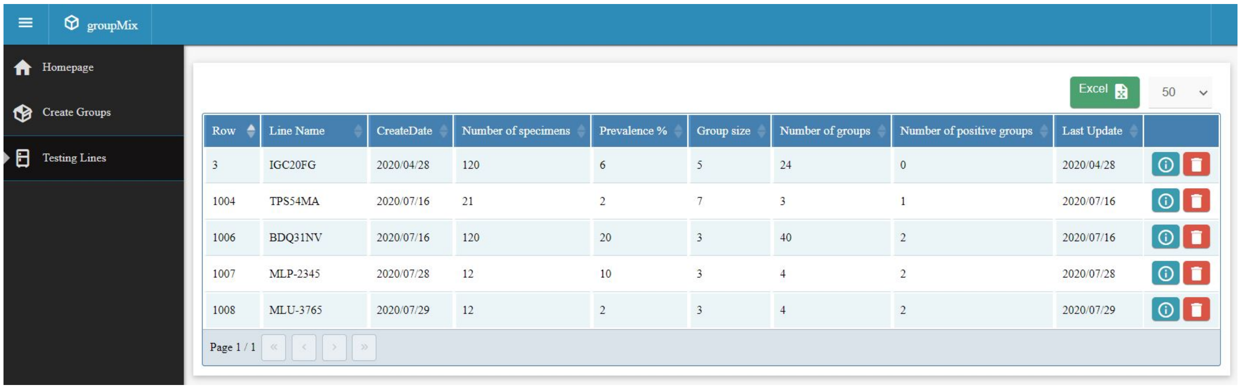

Fig. II.3. "Test lines" section in the groupMix web interface.
